# Pharmacological treatment of hypertension guided by peripheral or central blood pressure: a comparison between the two strategies

**DOI:** 10.3389/fcvm.2023.1247146

**Published:** 2023-09-13

**Authors:** Gilberto Campos Guimarães Filho, Priscila Valverde de Oliveira Vitorino, Sayuri Inuzuka, Adriana Sebba Barroso, Robson Pierre Pacífico Alves Filho, Victoria Alves Melo, Luiz Fernando de Oliveira Urzeda, Ana Luiza Lima Sousa, Antonio Coca, Paulo César Brandão Veiga Jardim, Weimar Kunz Sebba Barroso

**Affiliations:** ^1^ Hypertension League and Graduate Program, Department of Cardiology, Medicine School Federal University of Goiás, Goiânia, Brazil; ^2^ School of Social Science and Health, Pontifical Catholic University of Goiás, Goiânia, Brazil; ^3^ Hypertension and Vascular Risk Unit, Department of Internal Medicine, Hospital Clínic (IDIBAPS), University of Barcelona, Barcelona, Spain

**Keywords:** arterial stiffness, central blood pressure, clinical trial, hypertension, pulse wave velocity

## Abstract

**Background:**

Arterial hypertension treatment guided by central blood pressures (CPB) rather than peripheral blood pressures (PBP) measurement has the potential to show greater effectiveness in preventing or even regressing stiffness and target organ damage (TOD).

**Objective:**

This study aimed to compare the parameters of CBP and PBP measurements, arterial stiffness, TOD and renal profile in patients with anti-hypertensive treatment guided by CBP or PBP targets.

**Methods:**

A randomized clinical trial was conducted in central group (CG) and peripheral group (PG). Patients were randomized, evaluated every 3 months for BP and antihypertensive adjustments during a one-year follow up. The procedures in V1 and V5: anthropometric assessment; CBP/PBP measurements, carotid ultrasound; echocardiography; laboratory tests. Paired and unpaired *t*-tests and the *χ*^2^ were used (significance level: 5%).

**Results:**

The study evaluated 59 participants (30CG/29PG). The augmentation index (AIx) was higher in the CG (27.3% vs. 20.3%, *p* = 0.041). Intergroup analysis has found central diastolic BP lower in the CG (78.9 vs. 84.3 mmHg, *p* = 0.024) and the Alx difference between groups ceased to exist after a one-year follow-up. Intragroup comparisons, after intervention, showed a lower frequency of changed PWV (*p* < 0.001) and LVMI (*p* = 0.018) in the CG. The PG showed a higher frequency of changed PWV (*p* < 0.001) and LVMI (*p* = 0.003).

**Conclusion:**

The intervention guided by central BP reduced the central diastolic BP and AIx compared to the PG. There was a reduction in the frequency of changed PWV and LVMI in the CG.

## Introduction

Arterial hypertension (AH) is the main modifiable risk factor for cardiovascular disease (CVD) and premature mortality worldwide. It is traditionally diagnosed and treated based on peripheral blood pressure (BP) measurements (1–3).

The incorporation of arterial stiffness measurements into traditional scores for cardiovascular (CV) risk stratification and the early identification of vascular damage significantly improves the prediction of CV events. Pulse wave velocity (PWV) is a well-established measurement, an excellent biomarker that can identify subclinical target organ damage (TOD), and, when increased, is associated with a considerable increased CV mortality in hypertensive patients (1, 2, 4, 5).

The implementation of simplified technology and research on new low-cost methods to measure or estimate aortic stiffness have increased its use in clinical practice. Currently, different validated devices to measure central BP and PWV are available for clinical use (6) and can improve the prediction of a ten-year risk of CVD by 13% in intermediate risk patients (7).

Moreover, the presence of residual CV risk in the hypertensive population, delayed identification of subclinical damage, and implementation of optimized therapeutic strategies may be associated with difficulties in the absolute reduction of CV outcomes. The association of therapeutic strategies based only on peripheral BP measurements with these difficulties has been debated (3, 5, 8).

The hypothesis that the treatment guided by central BP reduction goals may present advantages over the conventional treatment strategy in reducing intermediate outcomes has biological plausibility (9–12). Few studies have tested this hypothesis, but the superiority of central BP parameters over peripheral ones in predicting CV risk highlights the importance of evaluating the possible behavior of some biomarkers such as PWV as risk factors (13–17).

Therefore, AH treatment guided by central BP parameters has the potential to show greater effectiveness in preventing or even regressing stiffness and TOD when compared to the conventional strategy (18, 19).

Thus, the objectives of this study were: (1) to verify if the treatment guided by central BP values has better effects on central BP values, carotid ultrasound, and Doppler echocardiography compared to the treatment guided by peripheral BP values; (2) to compare central BP values, carotid ultrasound, and Doppler echocardiography before and after the study in each of the groups; and (3) to compare inter- and intragroup frequency of changed PWV, left ventricular LVMI, and creatinine clearance.

## Patients and methods

This study is an open-label, randomized, clinical trial conducted in two AH reference services. The study protocol was approved by the Research Ethics Committee under opinion no. 2.746.523, and all participants signed the informed consent form before study procedures.

The inclusion criteria were patients with AH, aged 18 years or more, using or not using antihypertensive drugs, and with an indication for pharmacological treatment based on casual BP measurements (1).

The exclusion criteria were patients with end-stage chronic diseases or previous CVD, including coronary artery disease (acute myocardial infarction, angina, coronary artery bypass graft surgery, or angioplasty) or stroke (ischemic and hemorrhagic stroke or transient ischemic attack) less than six months before the study. These criteria were defined by information obtained directly from the patients or from complementary tests.

Study participants answered a sociodemographic questionnaire, had their body mass and height measured to calculate the body mass index (BMI) (20, 21), had their peripheral and central BP measured, and underwent Doppler echocardiography, carotid ultrasound, and laboratory tests.

Peripheral BP was measured in the office, in a quiet and silent environment, using an HEM-1100 OMRON® automatic device and following the recommended guidelines (1, 5). Central BP measurement was performed, under the same conditions, using the Cardios Dyna MAPA AOP® device with the ARV Solver algorithm (three consecutive measurement protocol and C1 calibration) to verify the central BP, PWV, total vascular resistance (TVR), and augmentation index (AIx).

Cardiac and vascular structural damage was assessed by Doppler echocardiography and carotid ultrasound using a TOSHIBA Xsario ultrasound device. The parameters analyzed included the interventricular septum and left ventricular posterior wall, LVMI, and left atrial volume measurement on Doppler echocardiography, and carotid intima-media thickness (cIMT) measurement and carotid plaque search on carotid ultrasound. All tests were performed by the same observer in each of the services.

The definition of cardiac and vascular damage was established using the following biomarkers: IMT >0.9 mm or presence of atherosclerotic plaques in carotid arteries (22, 23), left atrial diameter greater than 38 mm for women and 40 mm for men, LVMI >95 mg/m^2^ for women and >115 mg/m^2^ for men, and PWV ≥10 m/s (1, 5).

Creatinine was tested for the subsequent calculation of the glomerular filtration rate (GFR) using the Modification of Diet in Renal Disease (MDRD) formula and considering values ≤60 ml/min/1.73 m^2^ as reduced (24).

Treatment strategies were similar regarding the drugs used for both groups, and level adjustment was at the investigating physician's discretion to achieve the goals in both groups: level 1—Losartan 50 mg/day; level 2—Losartan 50 mg 12/12 hs; level 3—Losartan 50 mg 12/12 hs + Amlodipine 5 mg/day; level 4—Losartan 50 mg 12/12 hs + Amlodipine 10 mg/day; level 5—Losartan 50 mg 12/12 hs + Amlodipine 10 mg/day + Hydrochlorothiazide 12.5 mg/day; level 6—Losartan 50 mg 12/12 hs + Amlodipine 10 mg/day + Hydrochlorothiazide 25 mg/day; level 7—Losartan 50 mg 12/12 hs + Amlodipine 10 mg/day + Hydrochlorothiazide 25 mg/day + Spironolactone 25 mg/day.

There was no wash-out before randomization (1, 5, 6, 25). It is noteworthy that the use of the same antihypertensive drug strategy for both groups aimed to ensure that the only difference between them would be related to the goal guided by central or peripheral parameters.

After the initial visit, the participants were evaluated every 90 days to adjust the drug level. For the CG, the goal was to maintain central systolic BP below the values established with reference to sex and age group (25) (Table 1). For safety, the minimum limit for peripheral BP reduction was 110/70 mmHg.

**Table 1:** Central systolic blood pressure values according to age categories, for males and females, in the normal and reference populations (25).

	Normal population		Reference population	
	Female	Male	Female	Male
>20	97 (86, 91, 102, 109)	105 (95, 99, 109, 113)	99 (88, 93, 105, 120)	109 (96, 102, 117, 127)
20–29	95 (80, 88, 102, 110)	103 (92, 97, 109, 115)	101 (88, 94, 110, 124)	110 (95, 102, 120, 130)
30–39	98 (84, 90, 108, 119)	103 (88, 95, 112, 120)	111 (92, 100, 127, 141)	114 (95, 103, 129, 144)
40–49	102 (87, 93, 113, 123)	106 (90, 97, 114, 123)	116 (95, 104, 133, 146)	118 (97, 106, 132, 144)
50–59	110 (93, 100, 119, 127)	110 (96, 102, 118, 126)	120 (100, 109, 134, 148)	123 (102, 111, 137, 150)
60–69	114 (97, 105, 122, 129)	114 (97, 105, 122, 128)	128 (105, 115, 141, 154)	128 (105, 115, 142, 155)
70+	118 (100, 109, 126, 131)	116 (99, 107, 124, 130)	138 (113, 126, 152, 164)	135 (113, 124, 147, 160)

Values given here are 50th (10th, 25th, 75th, and 90th) percentiles.

For the PG, the goal was a peripheral BP value lower than 140/90 mmHg for low and medium risk and lower than 130/80 mmHg for high risk such as European Society of Cardiology and the European Society of Hypertension Guidelines (1, 5).

If the patient did not meet the defined goals in the return visits, the drug level was increased at medical discretion. Patients who did not show up for a visit after at least two contact attempts were considered lost to follow-up.

## Statistical analysis

Statistical analysis was performed with the Stata software version 14.0. The Shapiro Wilk test was used to verify the normality of data distribution. Quantitative variable values and deltas were compared between groups at the beginning and end of the study using the unpaired *t*-test for quantitative variables with normal distribution and the Mann–Whitney *U*-test for quantitative variables with non-normal distribution. The *χ*^2^ or Fisher's tests were used to compare qualitative sociodemographic, BP, and complementary test variables; drug level used at each visit; and the frequency of intragroup PWV, ventricular mass index, and creatinine clearance changes at the initial and final visits. The significance level was set at 5% for all tests.

## Result

The initial sample consisted of 130 participants, of whom 59 (30 CG and 29 PG) completed the study, with no deaths or serious adverse events. The 71 losses to follow-up (54.6%) occurred due to the coronavirus disease 2019 (COVID-19) pandemic.

At the initial visit, the groups were similar in terms of sociodemographic characteristics, BMI, cardiovascular risk factors, central and peripheral BP measurements, variables obtained by carotid ultrasound and Doppler echocardiography, and GFR. Only the AIx was higher in the CG (Table 2).

**Table 2:** Comparison between the central and peripheral groups before intervention regarding sociodemographic variables, body mass index, central blood pressure measurements, carotid ultrasound, Doppler echocardiography, and glomerular filtration rate, *n* = 59, 2018–2020.

Variables	CG (*n* = 30)	PG (*n* = 29)	*p*
Women	20 (66.7%)	20 (69.0%)	0.850
Age (years)	60.5 ± 9.8	59.1 ± 9.6	0.582
BMI (kg/m^2^)	29.2 ± 5.6	30.1 ± 5.2	0.507
Central BP			
Central systolic BP (mmHg)	125.0 ± 14.5	124.0 ± 14.6	0.793
Central diastolic BP (mmHg)	84.4 ± 10.6	86.5 ± 11.5	0.472
Peripheral systolic BP (mmHg)	133.4 ± 15.7	131.2 ± 14.9	0.574
Peripheral diastolic BP (mmHg)	83.5 ± 10.6	85.5 ± 11.6	0.481
Central pulse pressure (mmHg)	39.8 ± 8.8	36.2 ± 8.3	0.112
Augmentation index [AIx(%)]	27.3 ± 12.2	20.3 ± 13.3	**0** **.** **041**
TVR	1.3 ± 0.23	1.3 ± 0.20	0.176
PWV (m/s)	8.3 (8.0–10.3)	8.3 (7.7–9.3)	0.495
Carotid ultrasound			
Presence of plaque	14 (46.7%)	11 (37.9%)	0.497
Carotid IMT (mm)	0.8 ± 0.41	1.0 ± 0.23	0.053
Doppler echocardiography			
Interventricular septum thickness (mm)	9.0 (9.0–11.0)	9.0 (8.0–10.0)	0.543
LV posterior wall thickness (mm)	9.8 ± 1.33	9.5 ± 1.38	0.370
LV diastolic diameter (mm)	47.1 ± 4.7	47.4 ± 4.6	0.818
LV mass index (g/m^2^)	92.7 ± 27.5	88.9 ± 26.6	0.596
LA volume (ml/m^2^)	28.3 ± 6.4	30.0 ± 8.7	0.402
GFR (ml/min/1.73 m^2^)	75.0 ± 21.3	76.7 ± 19.9	0.746

GFR, glomerular filtration rate; LV, left ventricle; LA, left atrium; BMI, Body mass index; BP, Blood Pressure; IMT, Intima-Media Thickness.

*χ*^2^; Unpaired *t*-test; Mann–Whitney *U*-test.

No differences were identified in peripheral BP, carotid ultrasound and Doppler echocardiography variables, and in GFR after the 12-month follow-up. Central diastolic BP was lower in the CG than in the PG. The delta also showed a greater AIx and TVR reduction in the CG than in the PG (Table 3).

**Table 3:** Comparison of absolute values and deltas between the central and peripheral groups after the 12-month intervention regarding central blood pressure measurements, carotid ultrasound, Doppler echocardiography, and GFR, *n* = 59, 2018–2020.

	CG	PG	*p*	CG	PG	*p*
	Absolute values*			Delta*		
Central BP						
Central systolic BP (mmHg)	116.0 ± 2.8	120.0 ± 13.5	0.247	−9.0 ± 17.3	−4.0 ± 17.4	0.273
Central diastolic BP (mmHg)	78.9 ± 9.7	84.8 ± 10.4	**0** **.** **024**	−5.5 ± 8.6	−1.5 ± 12.2	0.151
Peripheral systolic BP (mmHg)	124.3 ± 14.1	128.0 ± 15.1	0.334	−9.2 ± 15.2	−3.2 ± 16.9	0.160
Peripheral diastolic BP (mmHg)	78.1 ± 9.8	83.1 ± 10.9	0.069	−5.4 ± 8.1	−2.4 ± 12.5	0.286
Central pulse pressure (mmHg)	36.5 ± 11.6	35.1 ± 9.5	0.624	−3.4 ± 14.4	−1.1 ± 7.9	0.466
Augmentation index [AIx(%)]	23.3 ± 11.0	23.8 ± 11.4	0.876	−4.0 ± 12.1	3.4 ± 12.1	**0** **.** **016**
TVR	1.3 ± 0.2	1.4 ± 0.2	0.311	−0.001 ± 0.2	0.13 ± 0.2	**0** **.** **038**
PWV (m/s)	8.3 (7.9–9.6)	8.4 (7.6–9.3)	0.785	−0.2 ± 0.6	0.1 ± 0.6	0.061
Carotid ultrasound						
Presence of plaque	12 (60.0%)	9 (47.4)	0.176	–	–	
Carotid IMT (mm)	0.7 ± 0.4	0.9 ± 0.3	0.237	0.06 ± 0.1	0.04 0.1	0.569
Doppler echocardiography						
IV septum thickness (mm)	8.7 ± 1.5	8.7 ± 1.4	0.943	−0.06 ± 0.9	−0.2 ± 0.7	0.108
LV posterior wall thickness (mm)	8.7 ± 1.4	8.8 ± 1.2	0.832	−0.6 ± 1.0	−0.2 ± 0.8	0.185
LV diastolic diameter (mm)	46.3 ± 3.4	45.9 ± 4.1	0.803	0.4 ± 2.1	0.2 ± 1.5	0.750
LV mass index (g/m^2^)	78.5 ± 26.9	71.0 ± 18.2	0.319	−1.6 ± 22.0	−8.1 ± 14.3	0.289
LA volume (ml/m^2^)	34.3 ± 10.1	33.4 ± 5.8	0.785	3.2 ± 8.5	−0.2 ± 8.0	0.272
GFR-MDRD (ml/min/1.73 m^2^)	74.55 ± 24.2	71.93 ± 20.3	0.657	0 -9–7	−5 -13–0	0.302

CG, central group; PG, peripheral group; BP, blood pressure; GFR, glomerular filtration rate, LV, left ventricle.

Unpaired *t*-test; Mann–Whitney *U*-test; Fisher's exact test.

**Figure 1:**
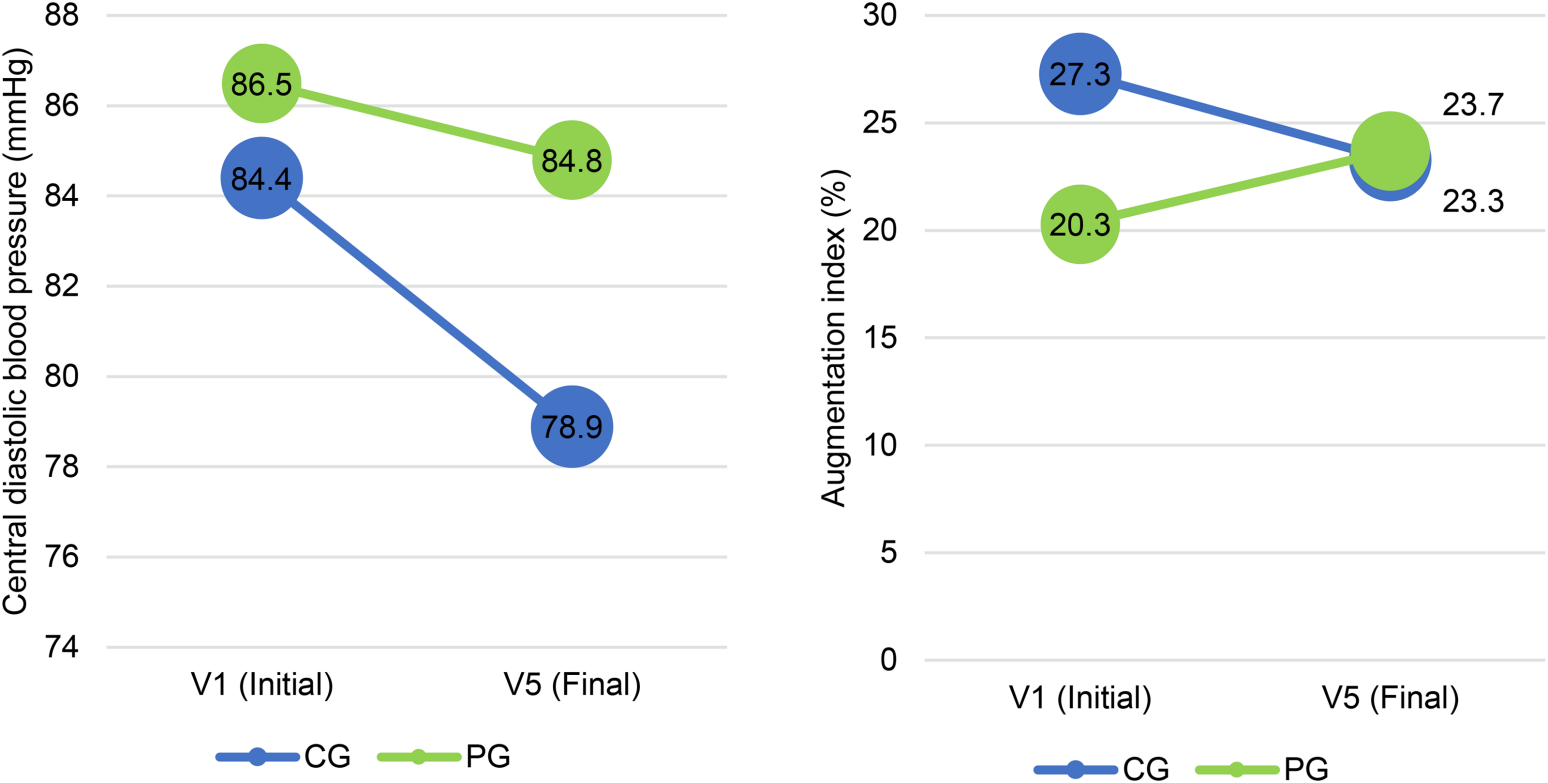
Central diastolic pressure and augmentation index values at V1 (initial) and V5 (final) in the CG and PG, *n* = 59. CG, central group; PG, peripheral group.

Central diastolic pressure and AIx were reduced in the CG and AIx was increased in the PG at the end of the one-year follow-up (Figure 1).

The use of three antihypertensive drugs showed a higher frequency than monotherapy and dual combination in both the CG and PG at the initial and final visits. There was no inter- or intragroup drug level difference before and after the follow-up (Table 4).

**Table 4:** Comparison of the number of drugs used before and after the intervention in the central (*n* = 30) and peripheral (*n* = 29) groups.

	Central group (*n* = 30)	Peripheral group (*n* = 29)	*p* (between groups)
Initial visit			0.222
Monotherapy	3 (10.0%)*	8 (27.6)*	
Double combination	9 (30.0%)*	7 (24.1%)*	
Three or more antihypertensives	18 (60.0%)^#^	14 (48.3%)^#^	
Final visit			0.111
Monotherapy	3 (10.0%)*	9 (31.0%)*	
Double combination	9 (30.0%)*	5 (17.3%)*	
Three or more antihypertensives	18 (60.0%)^#^	15 (51.7)^#^	

Comparison of intragroup treatment level at the initial and final visits: different symbols indicate statistical difference (*p* < 0.001).

There was no difference between the CG and PG regarding the frequency of changed PWV values (≥10 m/s), LVMI (>95 mg/m^2^ for women and >115 mg/m^2^ for men), and GFR (≤60 ml/min/1.73 m^2^) at the initial and final visits (Table 5).

**Table 5:** Comparison of the frequency of pulse wave velocity, left ventricular mass index, and glomerular filtration rate changes between the CG and PG before and after the study.

Variables	CG (*n* = 30)	PG (*n* = 29)	*p*
Initial visit			
PWV	8 (26.7%)	3 (10.3%)	0.108
LV mass index	10 (33.3%)	7 (25.0%)	0.486
Glomerular filtration	5 (17.2%)	4 (13.8%)	0.717
Final visit			
PWV	4 (13.3%)	4 (13,8%)	0.959
LV mass index	1 (5.3%)	2 (10.5%)	0.547
Glomerular filtration	8 (27.6%)	6 (20.7%)	0.539

Fisher's exact test.

When intragroup comparisons were made before and after the intervention, the CG showed a reduced frequency of participants with changed PWV (*p* < 0.001) and LVMI (*p* = 0.018). In the PG, the frequency of participants with changed PWV (*p* < 0.001) increased and of those with changed LVMI reduced (*p* = 0.003). The CG (*p* = 0.004) and PG (*p* = 0.004) showed increased frequency of changed GFR (Table 6).

**Table 6:** Comparison of the frequency of pulse wave velocity, left ventricular mass index, and glomerular filtration rate changes before and after the follow-up.

Variables	Initial visit	Final visit	*p*
CG			
PWV	8 (66.6%)	4 (33.4%)	<0.001
LV mass index	10 (90.9%)	1 (9.1%)	0.018
Glomerular filtration	5 (38.5%)	8 (61.5%)	0.004
PG			
PWV	3 (42.9%)	4 (57.1%)	<0.001
LV mass index	7 (77.8%)	2 (22.2%)	0.003
Glomerular filtration	4 (40.0%)	6 (60.0%)	0.004

Fisher's exact test.

## Discussion

Our study shows that the intervention guided by central BP reduced central diastolic BP but not central systolic BP, and corrected the AIx parameter after a one-year follow-up compared to the group guided by peripheral BP. Intragroup analysis showed a significantly reduced frequency of changed PWV and LVMI in the group of intervention guided by central BP.

This sample included hypertensive patients with a mean age of 60 years and mean BMI of 30 kg/m^2^ with well-controlled BP levels in the initial phase of the study. In addition, the comparative analysis in relation to the baseline characteristics showed that the groups randomized to treatment guided by central or peripheral pressure were similar, except for the AIx parameter, which was higher in the CG. As for the antihypertensive drugs used in our clinical trial, all patients used the same strategy in both groups to eliminate potential confounding factors that could occur in the case of different drugs. A recently published clinical trial randomized hypertensive patients to groups guided by the goal of PWV or peripheral BP reduction but used different classes and drugs in the follow-up phase (26).

PWV is considered an independent biomarker of subclinical TOD (27). To date, only the SPARTE study evaluated the strategy of AH treatment guided by PWV reduction compared with the strategy guided by peripheral BP and no significant differences were found to significantly CV outcomes, peripheral arterial disease, hospitalization for heart failure, aortic dissection, chronic kidney disease, and sudden death. However, the PWV guided treatment intensified the antihypertensive treatment with vascular aging prevention characterized by PWV behavior compared to the conventional treatment (26).

In our study treatment was guided with central or peripheral BP to achieve goals and considered PWV as an outcome variable. Although we found no significant difference between groups, the CG showed a significant reduction in changed PWV (≥10 m/s) over the 12-month follow-up. This finding corroborates the results of the SPARTE study and others that evaluated strategies to reduce vascular aging velocity (26–28).

In addition to PWV, our study also analyzed AIx, central BP, TVR, and central pulse pressure (CPP). We found a difference in central diastolic BP after a 12-month follow-up between the two randomized groups. Several studies evaluated these biomarkers, mainly as attempted surrogate outcome, and observed no significant association between central and peripheral BP measurements (29–32). However, a systematic review showed greater predictive power of central BP and CPP for TOD and CV outcomes (32). AIx was higher in the CG and reversed this behavior after the one-year follow-up.

Currently, evidence shows a stronger association between the central component of BP and increased LVMI and carotid IMT (33). Increased arterial stiffness is believed to be an intermediate stage between aging and CV damages, such as left ventricular and carotid dysfunction (34, 35). Our study showed no benefit in reducing CV outcomes such as left ventricular hypertrophy (LVH) and carotid vascular damage when treating hypertensive patients based on central BP compared with peripheral BP, and the frequency of subjects with changed LVMI reduced in both groups at the end of follow-up.

Two other studies that used the electrocardiogram as a measure of LVH showed a good association between central BP and LVH, but similar to that observed between peripheral systolic BP and LVH (36, 37). Another study reported the better predictive value of central BP compared to peripheral BP for cardiac damage such as LVH (38). A possible explanation is that arterial stiffness increases systolic BP, causing an early return of pulse waves during the systolic period and increased left ventricle afterload that causes cardiac hypertrophy and consequent LVH (39–41).

In our study, central BP had no better association with renal impairment than peripheral BP, a finding that corroborates with those of previous outpatient studies (42, 43). This may be justified by the fact that central BP is associated with macrovascular damage, but is not so closely related to microvascular injury, typical of renal injury (9). Another hypothesis suggests that in the early stages of kidney disease, the association between BP and kidney damage may be weak (44). We believe that longer follow-up and/or a larger sample size may detect greater CV protection with targets guided by central BP reduction.

The limitations of our study were mainly related to the loss of follow-up due to the COVID-19 pandemic, which may have impacted the statistical power to demonstrate differences between groups. Another limitation is related to a sample with well-controlled BP levels at the beginning of the study, which hinders differences in outcomes closely related to BP control. However, this is an unprecedented clinical trial in the comparison of goals guided by different strategies and with results that raise the need for further studies to provide the desired answers.

This clinical trial tested a hypothesis that is still under construction. Nevertheless, the intergroup difference in the behavior of AIx, central diastolic BP, and intragroup difference regarding the frequency of changed PWV and LVMI makes it plausible to consider the benefits of the treatment guided by central parameters.

## Conclusion

The treatment of hypertensive disease guided by central pressure reduction goals was not able to demonstrate differences in outcomes related to PWV, LVMI, IMT, and renal function compared to the traditional strategy, but showed superiority in reducing central diastolic pressure and AIx behavior at the end of a one-year follow-up. Intragroup analysis found a lower frequency of PWV ≥10 m/s in the CG, raising the hypothesis that longer follow-ups and greater sampling power may demonstrate the benefits of this treatment strategy.

## Data Availability

The raw data supporting the conclusions of this article will be made available by the authors, without undue reservation.
